# Trans-lateral ventricular approach for surgical treatment of high-located P2–P3 junction posterior cerebral artery aneurysms: from anatomical research to clinical application

**DOI:** 10.1007/s00701-024-05942-1

**Published:** 2024-01-30

**Authors:** Sajjad Muhammad, Rui Zhang, Timm Filler, Daniel Hänggi, Torstein R. Meling

**Affiliations:** 1 Department of Neurosurgery, Medical Faculty and University Hospital Düsseldorf, Heinrich Heine University Düsseldorf, Mooren Str. 5, 40225 Düsseldorf, Germany; 2 Department of Neurosurgery, King Edward Medical University, Lahore, Pakistan; 3 Department of Neurosurgery, Xingtai People’s Hospital Hebei Medical University, Xingtai, China; 4 Institute for Anatomy I, Medical Faculty and University Hospital Düsseldorf, Heinrich Heine University Düsseldorf, Mooren Str. 5, 40225 Düsseldorf, Germany; 5 International Neuroscience Institute (INI), Rudolf-Pichlmayr-Straße 4, 30625 Hannover, Germany; 6 Department of Neurosurgery, The National Hospital of Denmark, Rigshospitalet, Blegdamsvej 9, 2100 Copenhagen, Denmark; 7 Besta NeuroSim Center, Department of Neurological Surgery, Instituto Nazionale Neurologico “C. Besta,”, Milan, Italy

**Keywords:** Posterior cerebral artery aneurysms, Trans-lateral ventricular approach, Surgical clipping, Cerebrovascular disease

## Abstract

**Background:**

Posterior cerebral artery (PCA) aneurysms, though rare, pose treatment challenges. Endovascular therapy is the preferred option, but microsurgery becomes necessary in certain cases. Various microsurgical approaches have been suggested for PCA aneurysms, particularly those at the P2–P3 junction. This study highlights the trans-lateral ventricular approach (TVA) for addressing these complex aneurysms. This study aims to assess the feasibility and safety of the trans-lateral ventricular approach (TVA) for treating high-located complex PCA aneurysms at the P2–P3 junction. The study evaluates both clinical outcomes and anatomical considerations.

**Methods:**

Two cases of PCA aneurysms at the P2–P3 junction were treated using TVA in 2019. Navigation-guided entry via the interparietal sulcus was planned. Ventriculostomy was performed from the cortex to the lateral ventricle’s atrium. Medial atrial floor dissection exposed PCA’s P2–P3 segments. Neuronavigation and ultrasound-aided guidance was used. Anatomical studies on fixed and contrast-perfused specimens refined the approach.

**Results:**

Both cases saw successful aneurysm clipping. The unruptured aneurysm patient was discharged in 6 days. The poor-grade SAH patient required extended ICU care, moving to rehabilitation with mRS = 4. The unruptured complex aneurysm case exhibited no deficits, returning to work in 3 months. Anatomical dissections validated TVA for high-located P2–P3 junction PCA aneurysms.

**Conclusion:**

While endovascular therapy remains primary, this study demonstrates the viability of navigation-guided TVA for select high-located P2–P3 junction PCA aneurysms. Successes and challenges underscore the importance of patient selection and anatomical awareness.

**Supplementary Information:**

The online version contains supplementary material available at 10.1007/s00701-024-05942-1.

## Introduction

Posterior cerebral artery (PCA) aneurysms are rare, with an overall incidence of less than 1%, and only account for 7% of all posterior circulation aneurysms [1]. In cases of subarachnoid hemorrhage (SAH), the exclusion of aneurysms from the normal vasculature is crucial in the SAH treatment. In cases of incidental aneurysms, the growing and rupture-prone aneurysms in high-risk patients are recommended to be treated. Endovascular treatment (EVT), with the aim of occluding the aneurysm and keeping the PCA intact, is the first line of treatment for posterior circulation aneurysms [2].

Surgery is an alternative in cases of complex aneurysms that are difficult to treat with current endovascular means. Due to their deep location, PCA aneurysms are challenging to approach surgically. Depending on the exact location of the aneurysm, from the proximal to the terminal portion of the PCA, various surgical approaches have been proposed, for example, the pterional or the orbitozygomatic approach from the anterior direction, the sub-temporal approach from the lateral side direction, and the supra-cerebellar transtentorial approach from the posterior direction [3, 6].

The sub-temporal approach is the most commonly used approach for clipping PCA aneurysms. However, there are certain limitations to this approach, particularly if the aneurysm is high laying that needs retraction of the temporal lobe, as this can lead to iatrogenic damage to the parenchyma and major draining veins [7]. In this article, we demonstrate our novel approach for the high-located P2–P3 junction PCA aneurysm treatment that is based on extensive anatomical research and ultimately a safe clinical application.

## Methods and materials

### Anatomical research

**Fig. 1:**
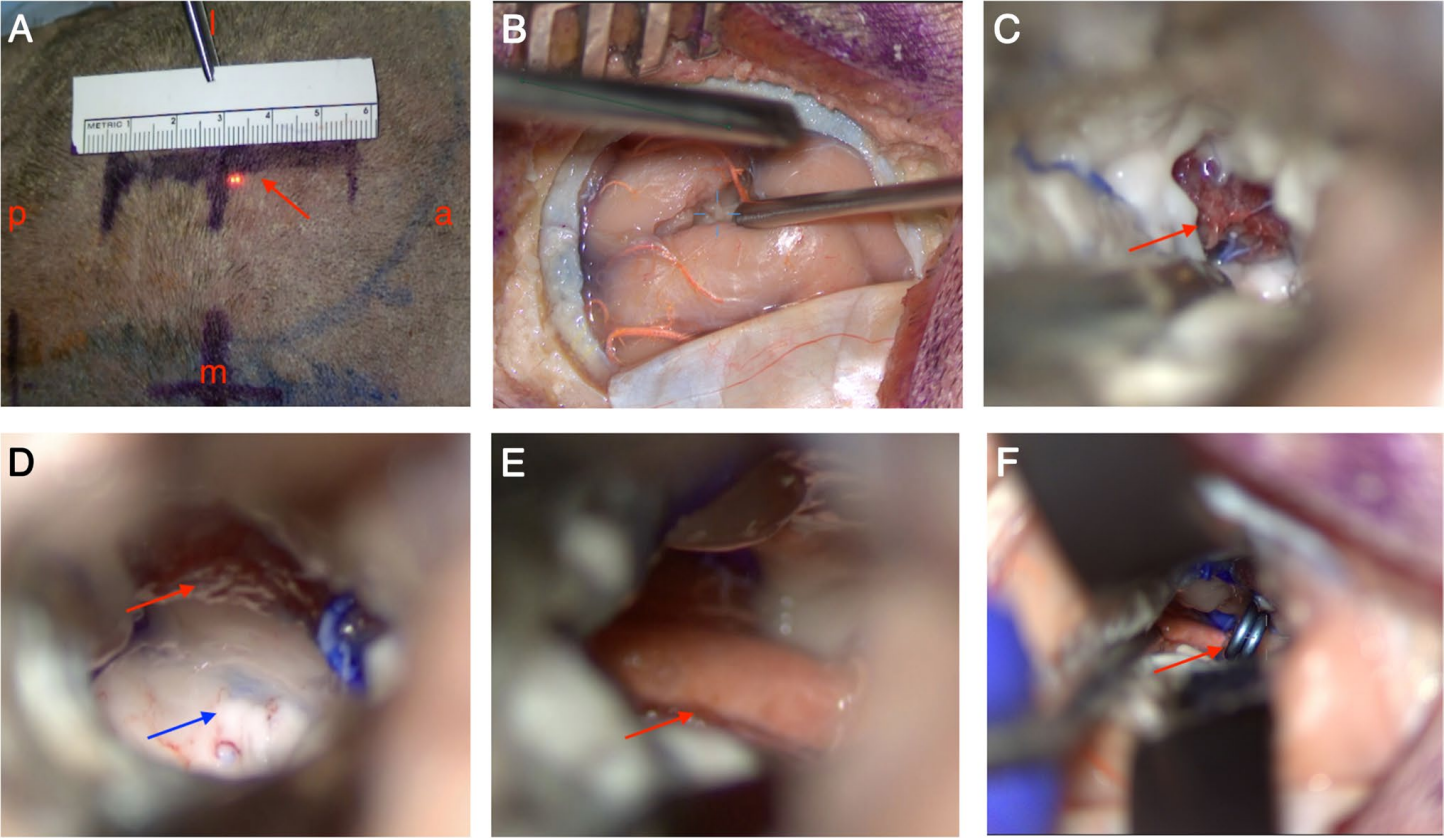
Step-by-step anatomical study on radiolucent contrast perfused cadaver. **A** The incision on the scalp; the red arrow show the incision position and its length. a, anterior; m, medial line; p, posterior; l, lateral. **B** The position of the cortical incision and corticotomy. **C** Entrance into the atrium portion of the lateral ventricle; the arrow pointing to the choroid plexus. **D** The red arrow show the choroid plexus, and the blue arrow point to the position of the corridor from the ventricle to the PCA site. **E**, **F** The red arrow shows the PCA segment and the clip on the PCA (P2–P3 junction)

A fresh skull specimen was perfused with a radiolucent contrast material and securely placed in a Mayfield head holder, followed by a highly detailed step-by-step imitation of a surgical procedure. After the preoperative CT angiography scan, neuronavigation was planned. The craniotomy was performed using a high-speed drill (Aesculap) and dissection was performed under high magnification using an operative microscope (Leica). The P2 and P3 segments of the PCA were exposed and a clip (Aesculap 720) was applied on the P2–P3 junction. The description of the surgical procedure is detailed in the surgical tech-note paragraph (Fig. 1).

### Clinical application

#### Case description

##### Case 1

**Fig. 2:**
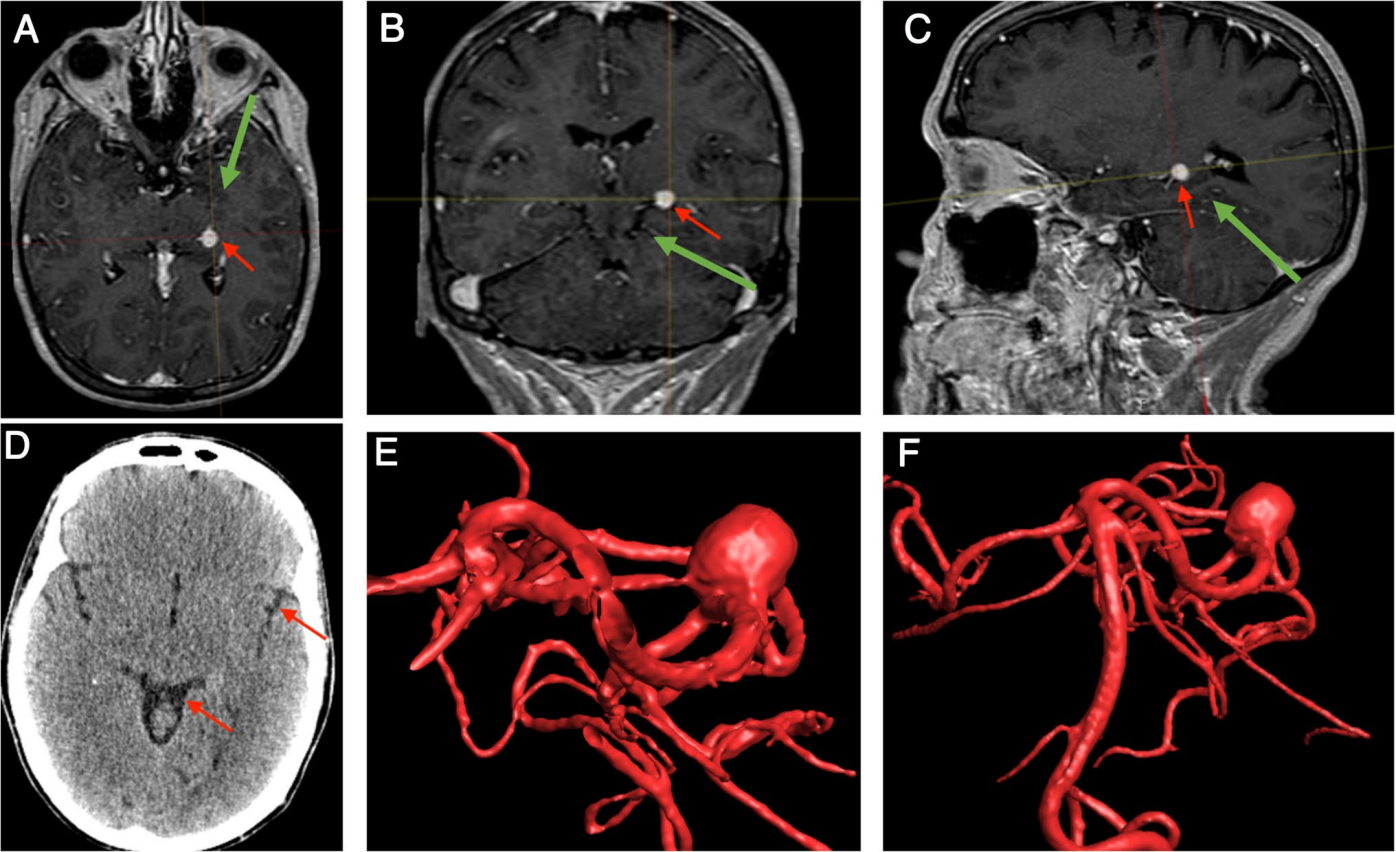
Preoperative images of case 1 with unruptured PCA aneurysm. The red arrows in A, B, and C point to the aneurysm position. The green arrows represent potential approaches to the aneurysm site: **A** pterional or orbitozygomatic approaches from the anterior direction; **B** sub-temporal approach from the lateral direction; **C** supra-cerebellar transtentorial approach from the posterior direction. **D** Red arrows show the cerebrospinal fluid in the Sylvian fissure and quadrigeminal cistern without any bleeding in basal cisterns. **E**, **F** Reconstruction of 3D angiography image from different angles shows a complex aneurysm with several branches

A 55-year-old female presented with symptoms of right-sided tinnitus and hearing loss in January, 2018. MRI showed an approximately 6.5 × 6.5 mm P2–P3 junction PCA aneurysm on the left side. One-year follow-up MRI scan showed a clear progression in aneurysm size and in December, 2019, the aneurysm had a complex configuration and measured 8 × 9 mm. The case was discussed at an interdisciplinary vascular board meeting and the treatment options were endovascular coiling with occlusion of the PCA or surgical clipping with a reconstruction of the PCA. The patient decided on surgery and underwent the surgery by applying the TVA approach (Fig. 2).

##### Case 2

**Fig. 3:**
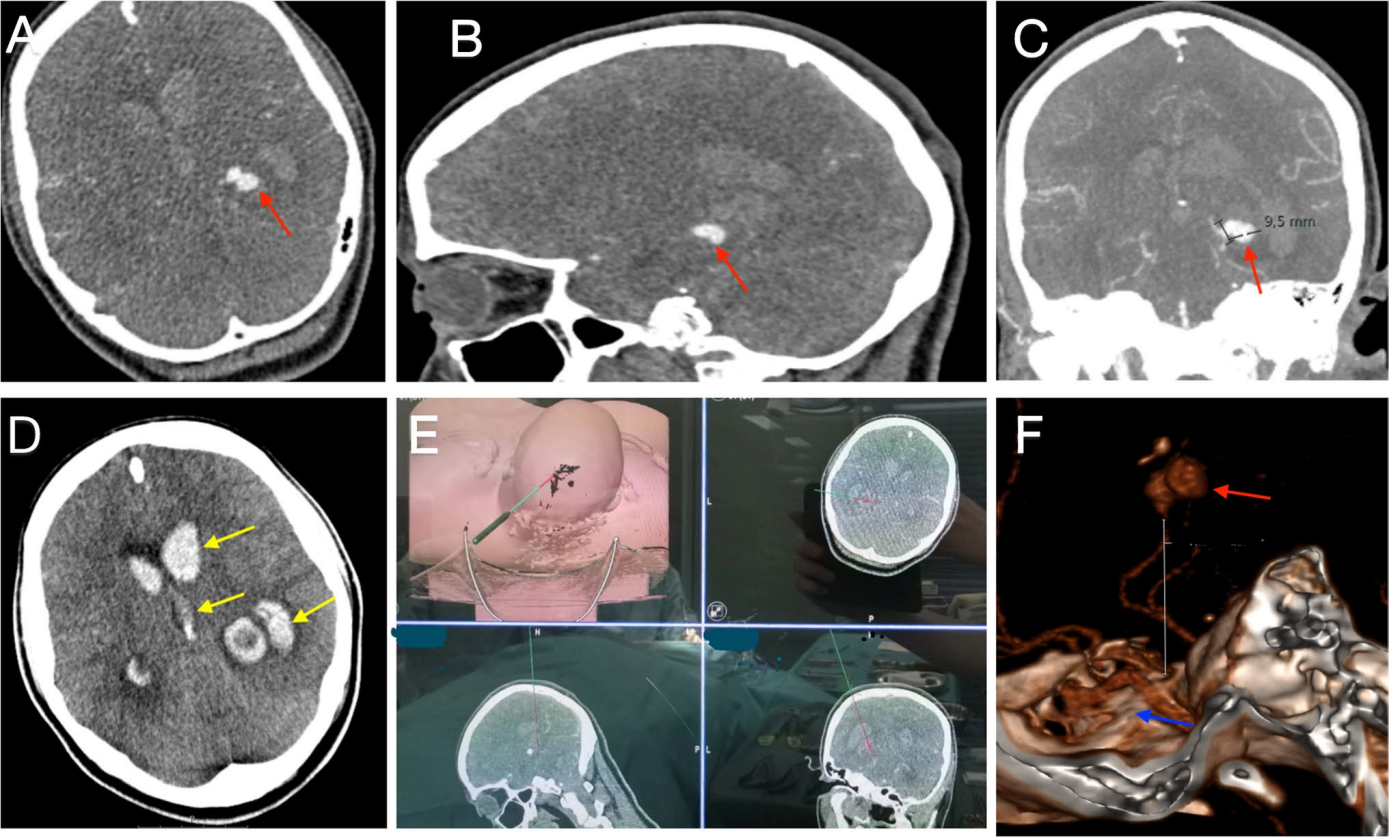
Preoperative images of case 2 with ruptured PCA aneurysm. The red arrows in A, B, and C point to the aneurysm position. **C** The diameter of the aneurysm (about 9 mm). **D** The yellow arrows show the hematoma in the lateral ventricles including the frontal horn, occipital horn, and the third ventricle. **E** Preoperative navigation planning used to design the surgical route based on CT data. Entry point and target can be observed before operation. **F** The 3D reconstruction image shows the aneurysm (marked with the red arrow) located high to the temporal skull base (marked with the blue arrow)

A 26-year-old female presented with a sudden onset of headache and general seizure. In our emergency room, intubation was performed due to a GCS of 4. Imaging by CT and CTA showed a SAH Fisher grade IV and a left-sided PCA aneurysm located at the P2–P3 junction. The patient was admitted to our neurosurgery department with SAH WFNS grade V. In the presence of SAH, intraventricular hematoma, and obstructive hydrocephalus, there was an emergency indication for EVD placement. After an interdisciplinary case discussion, we decided on surgical aneurysm clipping and hematoma removal. An emergency craniotomy was performed and the aneurysm was clipped via the TVA approach (Fig. 3).

The patient received informed consent for the operation and publication of data in the framework of our neurovascular research, and all the patients consented to the procedure and to the publication of his/her images.

#### Surgical technical note

##### Anatomical procedure

The specimen was fixed in the Mayfield head holder after performing a preoperative CTA scan. The data were imported into the BrainLab navigation system. An entry point in the parieto-occipital area was localized. The skin was incised approximately 6 cm long and was placed about 4 cm lateral to the med line at the parietal tuberentia about 6 cm above the inion. A standard burr hole was placed and a craniotomy was performed by using an Aesulap high-speed drill. The dura was opened under neuronavigation guidance and a corticotomy was performed in the interparietal sulcus. The brain parenchyma was dissected until the atrium portion of the lateral ventricle and the choroidal plexus could be identified. The floor of the lateral ventricle was pierced slightly posterior to the choroidal fissure, on the parahippocampal gyrus. The dissection should not be too close to the medial wall of the lateral ventricle, as this can cause damage to the body of the fornix and postoperative memory deficits. In our laboratory study, the PCA terminal segment in the cistern was successfully exposed, and an aneurysm clip was placed on the PCA (Fig. 1).

##### Clinical surgical procedure

After the successful induction of general anesthesia, the patient was positioned in a park bench position, and the head was fixed with a Mayfield head holder. A 5-cm-long skin incision was marked in the parieo-occipital area (focused on Frazier’s point) and the skin was disinfected and draped. The skin was incised to the bone and held with a skin retractor. A 3 × 3-cm craniotomy was performed just above the visual cortex, centered on the posterior horn of the lateral ventricle. The dura was opened, followed by a corticotomy and a trans-parenchymal dissection until the lateral ventricle was entered. The posterior commissure was identified using neuronavigation, and dissection of the commissure posterior to the choroid fissure continued until the PCA was located and the aneurysm was visualized. The aneurysm sac was dissected and the afferent and efferent vessels were identified. Temporary clips were placed on the supply vessels as well as the distal draining vessels and the aneurysm base was reconstructed using three clips (Aesculap FT710, 752, and 742). After the removal of the temporary clips, Doppler and ICG were used to confirm aneurysm obliteration and the integrity of the artery branches. A piece of collagen sponge was inserted into the parenchymal corridor, and a waterproof dural closure was performed to prevent postoperative CSF leakage. The bone flap was fixed and the skin closed as per standard procedure.

There were some modifications in the ruptured aneurysm case. A ventriculostomy was established and the hematoma was found in the lateral ventricle. During the procedure of removal of the hematoma, the aneurysm site was easily identified, as the high-pressure aneurysm bleeding had already destroyed the floor of the ventricle, and once we entered the ventricle, simply tracing the source of the hematoma located the aneurysm without additional removal of parahippocampal gyrus.

## Results

### Anatomical study

In our anatomical research, we found that it was not strenuous to create a corridor from the superficial cortex to the PCA located in the cistern, although the distance was approximately 8-cm deep. Every step could be identified by the neuronavigation; meanwhile, after entering the lateral ventricle, the choroid plexus and choroidal fissure could be utilized as landmarks (Fig. 1).

### Clinical case outcomes

In both clinical cases, the aneurysms were successfully clipped using a Brain Lab neuronavigation guidance.

**Fig. 4:**
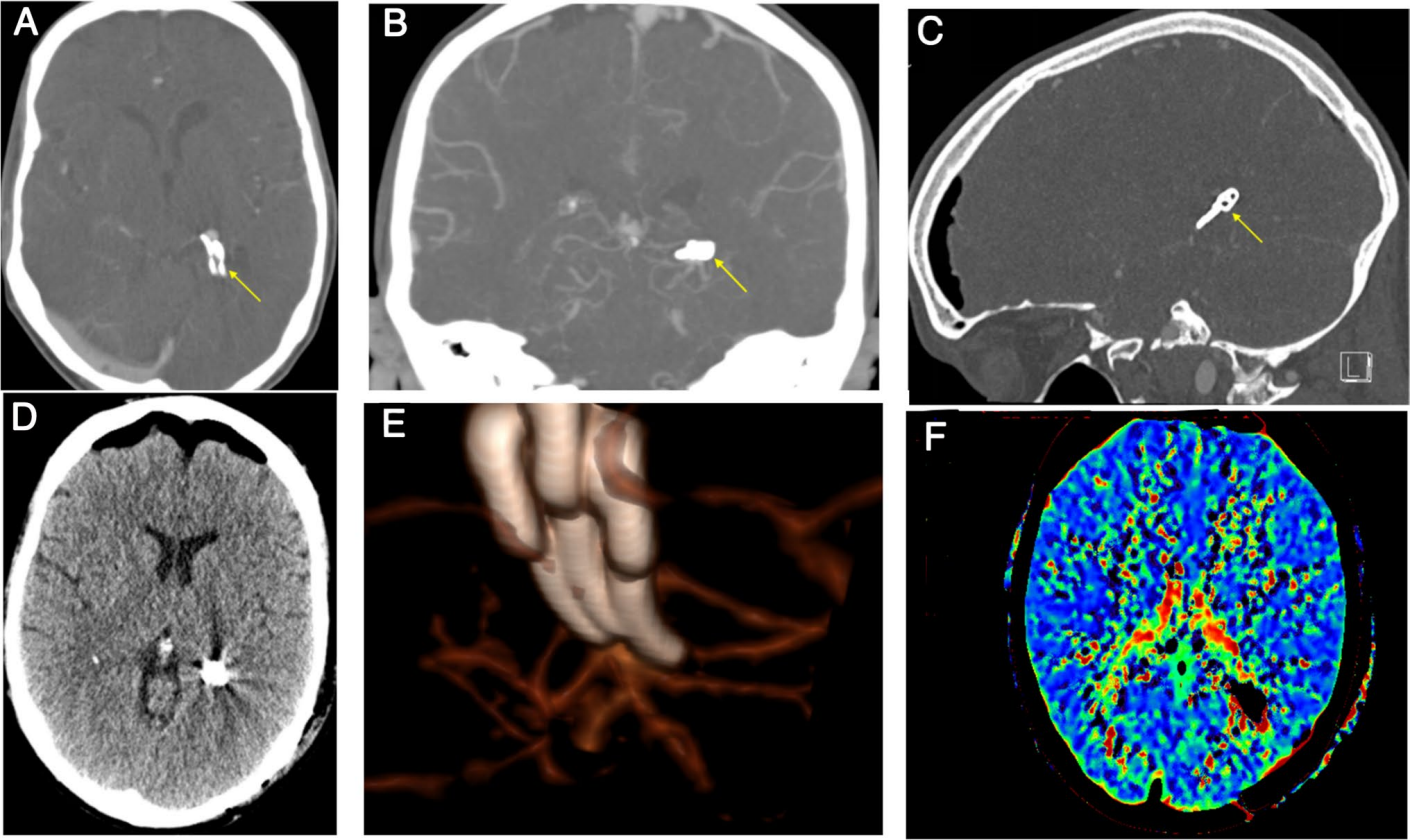
Postoperative images of case 1. **A**–**C** Axial, coronal, and sagittal CTA images show the position of aneurysm clips. **D** The postoperative CT scan show some postoperative intracranial air without any other complications. **E** Three clips were used to exclude the aneurysm and keep the patency of the vessels. **F** Postoperative perfusion CT scan shows that the area supplied by the PCA and its branches had a satisfactory outcome

Case 1: In this unruptured case, we utilized three clips to successfully reconstruct the intricate aneurysm, ensuring the continued patency of blood vessels and perforators. The patient experienced no postoperative complications and was discharged from our hospital within 6 days. During the 3-month follow-up, the patient exhibited no neurological deficits. Both the MoCA test at discharge and the one at the 3-month mark revealed no memory deficits. Remarkably, the patient had already resumed work by the 3-month follow-up (Fig. 4).

**Fig. 5:**
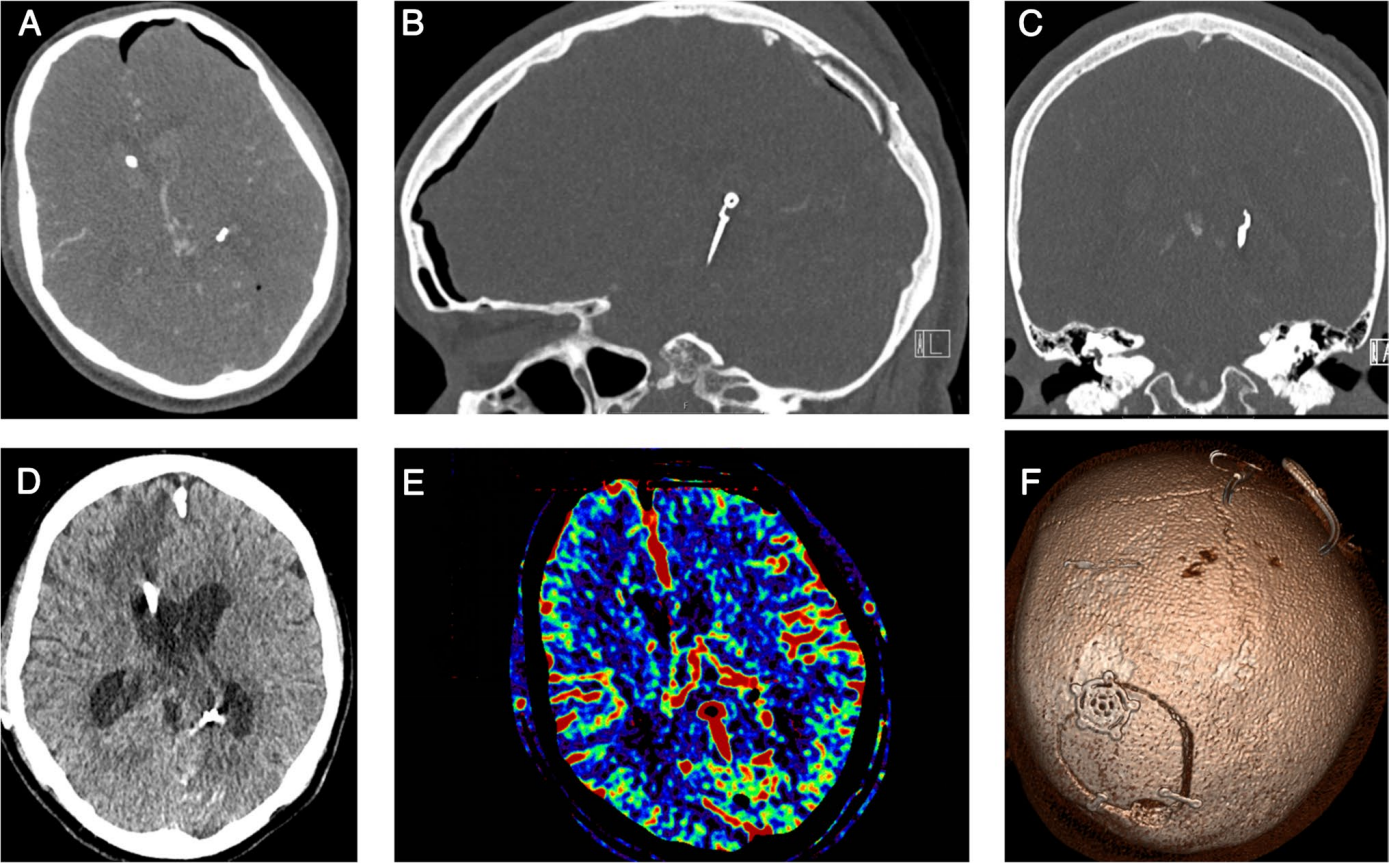
Postoperative images of case 2. **A**–**C** Axial, coronal, and sagittal CT images immediately after surgery demonstrating the clips’ position. **D** CT scans 5 days after surgery show the hematoma in the ventricles had disappeared. Red arrows show the EVD tube. **E** Postoperative CT perfusion scan shows no acute perfusion deficits. **F** 3D reconstructive image showing the craniotomy

Case 2: In this ruptured aneurysm case, the surgical procedure was performed safely, the patient required extended ICU care due to the poor preoperative SAH grade, and moving to rehabilitation with mRS = 4 (Fig. 5).

## Discussion

The posterior cerebral artery (PCA) is a terminal branch of the basilar artery that mainly supplies blood to the occipital lobe, the inferomedial surface of the temporal lobe, the midbrain, the thalamus, and choroid plexus of the third and lateral ventricles. The segmental anatomy of PCA is divided into five segments (P1 to P5). The P1 segment originates from the basilar tip and ends at the junction with the posterior communicating artery (PCOM). The P2 segment extends from the junction with the PCOM to the origin of the lateral temporal trunk at the level of the posterior part of the cerebral peduncle. The P3 segment extends from the origin of the lateral temporal trunk to the colliculi where the right and left PCAs are the nearest to each other (quadrigeminal point). It is located at the anterior-inferior side of the splenium. The P4 begins at the quadrigeminal point and ends at the top of the cuneus. While the P5 segment is named the terminal branch of the major terminal branches of the PCA, no definite limit has been described between the P4 and the P5 segments [8]. The above-mentioned classification is more appropriate for radiological and anatomical purposes, but at least the P1 to P3 definitions are also suitable for surgical purposes.

PCA aneurysms can be found along the whole course of the artery, but the most frequent locations are the P1 segment, the P1/2 junction, and the P2 segment. Goehre et al. [5] analyzed 34 patients with 37 PCA aneurysms and found that most aneurysms were located at the P1 to P3 segments (P1 segment *n* = 6; P1–P2 junction *n* = 10; P2 segment *n* = 16; and P3 segment *n* = 5). Only a few PCA aneurysms are located distal to P3.

Endovascular treatment (EVT) of aneurysms has developed rapidly with respect to technique and coiling materials. Most aneurysms situated on the posterior Willis circle, including PCA aneurysms, are treated by coiling, stent-assisted coiling, or flow diverters [2, 9–11]. However, despite great developments in EVT, some complex PCA aneurysms still need open surgery, in particular, aneurysms with complex branching, fusiform shape, or involvement of brainstem perforating branches [12].

### The surgical strategies and potential approach for PCA aneurysm treatments

The PCA aneurysm configuration and location in relation to skull base structures are particularly important when choosing the microsurgical approach [13]. The location of aneurysms at a particular segment dictates the surgical approach. The anterior approach, such as via pterional or orbitozygomatic craniotomies, is practical when dealing with P1 segment aneurysms. The posterior interhemispheric approach is suitable for reaching terminal PCA aneurysms. The supra-cerebellar transtentorial approach with patients in the sitting position is useful for P2 and P3 aneurysms [5, 6]. The lateral or sub-temporal approach is the most common route to the tentorial margin and interpeduncular cistern, which was proposed for the first time by Drake in 1961 for the treatment of basilar aneurysms [3]. Later, Drake and other neurosurgeons adopted this approach for the treatment of PCA aneurysms [4]. The greatest benefit of the sub-temporal approach is that in this route, the natural corridor between the temporal lobe and the middle cranial fossa is well utilized without any sacrifice of the brain cortical parenchyma. The P1 segment, the P1/2 junction, the P2 segment, and the anterior part of the P3 segment of the PCA can often be exposed using the sub-temporal approach [14, 15]. Meanwhile, as retraction of the temporal lobe cannot be infinite, some high-located PCA aneurysms may not be safely reached via this approach, such as distal PCA segment (distal than P3) or P1, P2 aneurysms higher than the posterior clinoid process [4–6].

### The trans-lateral ventricular approach (TVA) for treatment of high-located P2-P3 junction PCA aneurysms

Based on previous research, the approach to the atrium of the lateral ventricle is well established and used in routine practice [16]. In fact, the P2 segment is hidden under the parahippocampal gyrus as it winds around the side of the midbrain in the ambient cistern. Occasionally when performing the sub-temporal approach, it is necessary to remove around 1 cm of the gyrus in order to expose a highly placed or more complex aneurysm lying on the upper midbrain in the choroidal fissure [5]. The dissection of the parahippocampal gyrus during the subtemporal approach is performed from the inferior to the superior direction. In the TVA approach, the part of parahippocampal gyrus is dissected from the floor of the lateral ventricle toward the PCA which is a much shorter distance to reach the PCA. Neuronavigation is particularly helpful to correctly localize PCA if the landmarks are not clearly defined.

### Advantages and limitations of TVA and compression with other approaches

**Fig. 6:**
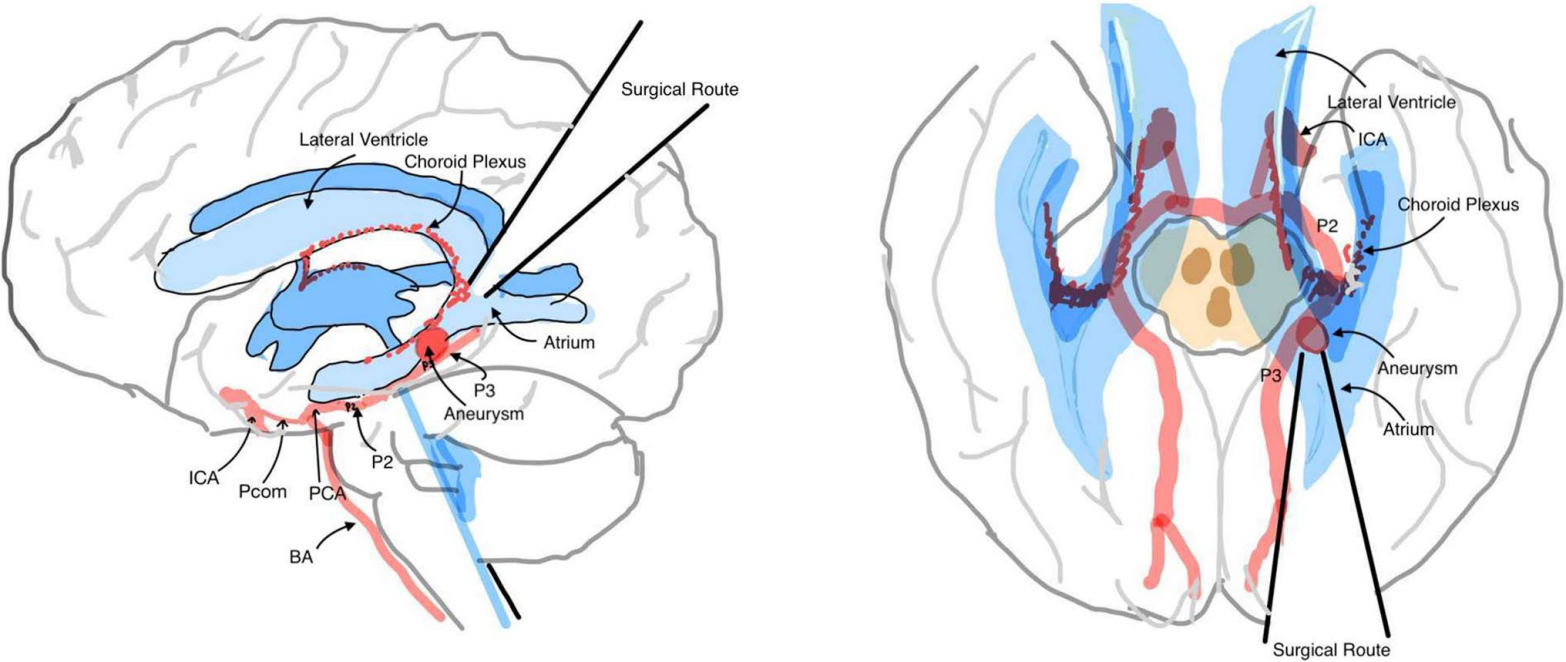
Overview schematic of the TVA surgical route. **A** Sagittal view illustrating the TVA surgical route. **B** Axial view of the TVA surgical route. Both showcase a direct trajectory from the cortex surface to the aneurysm site via the atrium without any sight obstacles

The TVA provides a straight route from the brain surface to the PCA aneurysm site (Fig. 6). In the dissection process, only cortical tissue is removed to approach the ventricle, and this region is considered a relatively “non-eloquent.” Most importantly, there is no exposure of any cranial nerves during this approach and hence no risk of any cranial nerve deficits, which is the second most common complication in the sub-temporal approach [5, 17].

Cerebrospinal fluid (CSF) release is the critical step during aneurysm surgery, particularly in the sub-temporal approach. The key step of the sub-temporal approach is to release the CSF preoperatively through the spinal lumbar drainage, and a slack brain plays an essential role in the elevation of the temporal lobe, to avoid lobe contusion or damage to the vein of Labbé [7]. In contrast, in the TVA, no CSF release is needed before the operation and, in fact, too much CSF loss during the surgical procedure will cause the mobility of the brain tissue, which has a negative effect on the precision of the neuronavigation.

Case selection is a significant factor in the success of surgery. In our opinion, the distance of the aneurysm to the floor of the atrium and the direction of the aneurysm dome should be taken into careful consideration preoperatively. TVA is a beneficial complementary to the high-located PCA aneurysm treatment.

## Supplementary Information

Below is the link to the electronic supplementary material.

Supplementary file1 (MOV 276560 KB)

Supplementary file2 (MOV 479954 KB)

## Data Availability

All data and materials used in this study are available upon reasonable request. Please contact Sajjad Muhammad at sajjad.muhammad@med.uni-duesseldorf.de for any inquiries regarding the availability of data and materials.
